# Management of Brain Arteriovenous Malformations: A Review

**DOI:** 10.7759/cureus.34053

**Published:** 2023-01-22

**Authors:** Nitesh Naranbhai, Raúl Pérez

**Affiliations:** 1 Department of Neuroscience, School of Advanced Education Research and Accreditation (SAERA), Universidad Isabel I, Burgos, ESP; 2 Department of Neurosurgery, Robert Mangaliso Sobukwe Hospital, Kimberley, ZAF

**Keywords:** multi-modality, embolization, radiotherapy, cerebral arteriovenous malformations, cerebral av malformation, bavm, avm, brain arterio-venous malformation, brain av malformation, av malformation

## Abstract

Brain arteriovenous malformations (bAVM) are vascular malformations of the brain affecting all ages. The optimum management strategy is essentially devoid of high-quality evidence and is highly nuanced and embedded in local customs. This study summarizes the frequently employed management strategies, drawing conclusions on the utility of each method of treatment and delving into controversies surrounding them. A literature search on PubMed and Medline was done on January 3^rd^, 2022. 11,767 articles were found, and abstracts were reviewed. Full-text review of 153 articles led to chapters from three books and 71 articles incorporated into a summative discussion. Spetzler-Ponce (S-P) Class A patients may be offered surgery if they are good surgical candidates and have a good number of high-quality years of life left. The exception is diffuse Spetzler-Martin (S-M) grade 2 in a patient older than 40 years: radiosurgery for unruptured and embolization for ruptured. S-P Class B may be offered surgery if a compact nidus or if younger than 40 years. If diffuse or age greater than 40, radiosurgery may be preferred if the Pollock-Flickinger score is less than 2.5. For the remainder of S-P Class B, conservative management may be preferred. S-P Class C is generally not treated unless young or those patients with poorly controlled seizures affecting their quality of life are willing to risk permanent neurological deficits. While the quality of studies is generally high, the level of evidence is concerning with only one randomized controlled trial (RCT). Most research output hails from high-income countries, i.e., perhaps not universally applicable to all settings owing to possible genetic, environmental, and resource differences. More research is needed: large volume studies in the pregnant population, validation of scoring systems in pediatric age groups, clinical trials focused on combination multi-staged treatment modalities, and studies originating from the developing world.

## Introduction and background

Brain arteriovenous malformations (bAVM) are a low prevalent important cause of intracranial bleeding, especially in young patients. bAVM can be defined as a dysplastic vascular nidus with feeding arteries and draining veins, comprising a conglomeration of numerous arteriovenous shunts without interposed brain tissue and no capillary bed [1]. The absence of resistance offered by capillary beds results in a state of increased blood flow, ultimately forming a high-flow arteriovenous shunt. Over time, this high-flow shunting will result in structural changes in vessel walls, even if feeding arteries and draining veins may have previously been normal [2]. Structural changes in feeding arteries include dilation, smooth muscle hyperplasia, and ingrowth of fibroblasts and connective tissue elements, forming fibromuscular cushions [3]. Changes in the venous system also include dilation and thickening of draining veins, the so-called “venous arterialization” [1]. Recent reports indicate that stenosis of the draining vein at or near the venous-dural sinus junction raises pressure within the nidus, increasing laminar wall shear stress throughout the bAVM, leading to inflammatory and degenerative changes and increasing the risk of rupture [4]. Although this stenosis has been found to progress with age and other vascular risk factors [5], this has yet to be confirmed in prospective data.

Etiology is classically described as congenital and associated with some syndromes in approximately 2% [3]. In Osler-Weber-Rendu syndrome, also known as hereditary hemorrhagic telangiectasia, often multiple bAVMs are seen, more commonly within families [6]. Other syndromes associated with bAVM are Cobb syndrome and cerebrofacial arteriovenous metameric syndromes involving the face and brain, such as Wyburn-Mason syndrome and Bonnet-Dechame-Blanc syndromes [7]. However, sporadic cases acquired after birth [8] are by far the most common, with a global prevalence of 0.04% to 0.52% [9] and may be due to upregulation or downregulation of multiple homeobox genes involved in angiogenesis, for example, HOXD3 [10] and HOXB3 [11]. Whole-exon sequencing in murine models has confirmed that vascular endothelial growth factor (VEGF), VEGF receptor, bone morphogenic protein, and transforming growth factor beta play a significant role in the pathogenesis of bAVM [3]. In addition, a high prevalence of KRAS/BRAF mutations has been found, raising the possibility of a therapeutic target in the distant future [3].

Based on location, bAVM may be classified as parenchymal, dural, or both. The parenchymal may be subclassified into pial, subcortical, paraventricular, or combined [8]. Spetzler-Martin (S-M) [12] have graded bAVM based on size (1, 2, or 3 points for <3cm, 3-6cm, or >6cm), relation to the eloquent cortex (1 point if eloquent area, i.e., sensorimotor, language, visual cortex, hypothalamus, thalamus, internal capsule, brainstem, cerebellar peduncles, deep cerebellar nuclei) and type of venous drainage (1 point if deep system, i.e., internal cerebral vein, basal vein, pre-central cerebellar veins). Each point indicates a grade, and a separate grade 6 is reserved for untreatable lesions by any means.

There are concerns about the suitability of S-M grading in the pediatric population as bAVMs only mature at about 18 years of age, tend to become more compact with time, and possible inter-observer reliability of assigning points [13,14]. However, the S-M grading is highly validated for estimating the risk of surgical resection [15]. The most common grade to present to academic centers is grade 3 [16]. The S-M grade has subsequently been condensed into a Spetzler-Ponce (S-P) class system to assist in management recommendations [17]. S-M grades 1 and 2 are S-P class A, S-M grade 3 is S-P class B, and S-M grades 4 and 5 are S-P class C. The fourth commonly used classification system is the Lawton-Young (L-Y) grading scale [18] using age (1 point if <20 years, 2 if 20-40 years and 3 points if >40 years), presentation (1 point if ruptured), a pattern of venous drainage (1 point if diffuse nidus). The combination of the S-M grade and L-Y scale forms a fully weighted model that can be used when there is a mismatch between the S-M grade and the L-Y scale.

Overall prevalence is reported as 0.14% and is detected in 0.51-1.3 per 100 000 person-years [3,15]. With a slight male preponderance [8,19], most are symptomatic between the ages of 20 and 50 [3], with 64% of bAVM diagnosed before the age of 40 [8]. In children outside the neonatal period, bAVM is the most common cause of spontaneous brain hemorrhage [20]. Presenting symptoms are hemorrhage in 65% [21], seizures in 15%-35% [3,15], and 8% presenting with either mass effect, ischemic features (steal phenomenon), headache or migraines, bruit (more common with dural bAVM), or raised intracranial pressure (ICP) [8]. Additional uncommon features in the pediatric population include hydrocephalus with macrocephaly (due to compression of aqueduct of Sylvius by raised ICP or by an enlarged vein of Galen in large midline bAVMs), congestive heart failure, and prominence of forehead veins (due to raised venous pressure) [8].

Data regarding hemorrhage are mainly from observational studies, so the true natural history of conservative management is unknown [22]. Approximately 38% of cases of non-traumatic intra-cerebral hemorrhages in those aged between 15 and 45 years are due to AVM rupture [23]. Location of hemorrhage is most commonly intra-parenchymal (ICH) in about 82% [24], followed by intraventricular extension of intra-parenchymal, pure intra-ventricular (IVH) (due to intra-ventricular located bAVM), subarachnoid (SAH) (likely due to aneurysm rupture), and uncommonly subdural (SDH).

The mechanism of seizure generation is thought to be due to ischemia from arterial steal or hemorrhage or due to localized irritation from mass effect [25]. Focal seizures are more common than generalized [3]. Risk factors for seizures are location (90% of bAVM presenting with seizures are supratentorial), lack of prior hemorrhage, and large nidus [26]. Headache in the absence of hemorrhage may be secondary to a primary underlying headache disorder or due to bAVM venous outflow obstruction [27] or prolonged meningeal artery steal phenomenon [3]. Neurological deficit without rupture (10% of bAVM) may be caused by the steal phenomenon, micro-hemorrhages, mass effect, or hydrocephalus [27].

Initial management of ruptured bAVM is in accordance with the 2022 American Heart/Stroke Association spontaneous ICH management guidelines [28]. Definitive management of ruptured bAVM may be simplified according to the S-P 3-tiered class: class A benefiting from surgical resection unless elderly or diffuse, class B multi-modality treatment using L-Y scales, and for class C observation with repeat angiogram every five years unless progressive neurologic deficit, steal-related symptoms or associated aneurysm is identified [17]. Management of unruptured bAVM has been a long source of controversy as morbidity associated with treatment may be more significant than the natural history. Options include conservative, open surgery, microsurgery, radiation as single-stage or multistage, and embolization. To add to the complexity and controversy, these treatment methods may be combined.

There is limited high-class evidence to aid in the decision algorithm, and current treatment is guided by anecdotes, case reports and series, a few systematic reviews, and only 1 “gold standard” randomized controlled trial, the ARUBA trial [29], which failed to identify outcomes of different management pathways. Thus, management remains somewhat nuanced and embedded in local customs. The objective here is to investigate the nature of the evidence for different arms of treatment of both incidental and symptomatic bAVM, summarize the management strategies currently employed and delve into a discussion regarding the controversies surrounding them.

## Review

Materials and Methods

Medline and PubMed were searched on the 3^rd^ of January 2022 using the following search terms: (((brain) OR (cranial) OR (cerebral)) AND ((avm) OR (av malformation) OR (arteriovenous malformation) OR (arterial venous malformation) OR (arterio-venous malformation)) AND ((management) OR (treatment) OR (therapy)). Abstracts were scanned for suitability. Inclusion criteria included systematic reviews, meta-analysis, randomized trials, cohort studies, case series of more than 10 patients, articles that have changed management as per referencing in any existing guidelines, and other articles deemed suitable for statistical referencing. Exclusion criteria were articles not in English, grey literature, ongoing trials, reports with data not reliably extracted, duplicate data, overlapping data, abstract-only papers as preceding papers, conference, editorial, and author response theses, and articles with no full text available. Abstract review led to the selection of articles. Full-text versions and chapters from selected books were incorporated into the discussion.

Results

A total of 11 767 articles were found, abstracts reviewed, and inclusion-exclusion criteria applied, resulting in 153 full-text reviews. Finally, 71 articles and chapters from three books were utilized for discussion. In Table 1, selected articles are summarized, with the key variable listed in the center, followed by key findings that had a great impact on our current management strategies.

**Table 1:** Summary of results of selected articles

Reference	Variable Studied	Result
Alqadi et al., 2019 [4]	Venous stenosis at draining vein-sinus junction	May contribute to brain arteriovenous malformation formation and rupture.
Al-Shahi et al., 2002 [13]	Observer agreement in angiographic assessment	Angioarchitectural features reported are inconsistent.
Bateman et al. 2006 [30]	Intracerebral haemorrhage in pregnancy	No increased risk of haemorrhage in pregnancy.
Bervini et al., 2014 [31]	Unruptured – surgery is better than conservative in selected case	Adverse outcomes for surgery are 10% and 18% for class A and B – better outcome than 5- and 10-year natural history, respectively.
Davidson et al., 2010 [32]	Safety of surgery as first-line treatment	Higher risks associated with surgery in certain locations, deep venous drainage, size.
Gross et al., 2012 [33]	Haemorrhage in pregnancy	Increased risk of rupture in pregnancy.
Gross et al., 2013 [34]	Meta-analysis on natural history	Risk of hemorrhage greater in previously ruptured (4.5% versus 2.2% for unruptured), deep venous drainage, deep location, associated aneurysm.
Iancu-Gontard et al., 2007 [14]	Variability in assessment of angiographic features	Angioarchitectural features reported are inconsistent.
Jayaraman et al., 2007 [35]	Haemorrhage rates in Spetzler-Martin grade IV and V	Increased risk with higher grades.
Kano et al., 2012 [36]	Radiosurgery	Planned multi-staged treatment, including focused irradiation had more deaths than cures.
Khaw et al., 2004 [37]	Association of infratentorial location with haemorrhage	Posterior fossa is higher risk.
Kim et al., 2014 [38]	Haemorrhage predictors in untreated	Previous rupture strongest predictor (4.3% versus 1.3% unruptured). Other predictors are age (30% increased risk per decade), exclusive deep venous drainage (1.6-2.4 x increased annual risk), aneurysm (for the first year).
Kim et al., 2015 [39]	Validation of Spetzler-Martin grading system	The use of Spetzler-Martin grade alone to determine recommendation for surgery should be cautioned.
Lawton et al., 2010 [18]	A supplementary grading scale	Lawton-Young classification system.
Liu et al., 2014 [40]	Risk of rupture during pregnancy	No increased risk.
Mohr et al., 2014 [29]	Randomized trial of unruptured medical management with or without intervention	Embolization followed by radiosurgery found treatment to be worse than no treatment.
Morgan et al., 2017 [41]	Review and natural history	Pre-operative embolization is not a universal practice.
Patel et al., 2019 [42]	Surgery for low grade by early career neurosurgeons	Cumulative summation method to assess impact.
Pollock et al., 2008 [43]	Modification of radiosurgery-based grading system	Prediction of radiosurgery deficit without obliteration can be estimated using the Pollock-Flickinger formula.
Spetzler et al., 1986 [12]	Grading system	Spetzler Martin grading system.
Spetzler et al., 2011 [17]	3-tiered classification	Spetzler Ponce class system.
Stapf et al., 2006 [44]	Predictors of haemorrhage in untreated	Associated aneurysm have higher risk

Discussion

Patient-centered counseling is difficult with bAVM. Discussion must negotiate between risks from intervention versus the natural history of the disease. When discussing complication rates, providing a single-point estimate based on benchmark results obtained at treating centers may not be appropriate as results vary between an early-career solo-practicing neurosurgeon versus an experienced neurovascular surgical team in a high-volume academic center. Ideally, outcomes at treating centers should undergo periodic audits to assess the impact of any new surgeon, and if such an audit is not possible due to low prevalence, perhaps there is not enough experience at that center to warrant definitive management. An alternative is the Cumulative Summation method [42]. A value is assigned to success (+0.967) and failure (-0.033) after each operation, and is added to the “unacceptable boundary value” as determined by 95% confidence intervals from the literature. The plot of sequential summation of outcomes must then fall within the pre-set acceptable boundary benchmark, allowing for comparison and perhaps a more appropriate quote of outcomes and risks. Another concern is the generalizability of results. One must be sure that the population characteristics and resources available are similar to studies quoted, including correction for biases that may have arisen from the untreated cases excluded from the results of some studies.

There are several reasons why the natural history of bAVM is fraught with discussion. bAVM has a low prevalence of no more than 1.5 per 100 000 people [15,45]. Angioarchitectural features reported are inconsistent, making the comparison a challenge [13,14]. There may be bias inherent to research from clinical databases [46]. The overall annual risk of future hemorrhage is between 2% [47] and 4% [48] and may be impacted by a large number of variables, including previous rupture (the strongest predictor, 4.3% annual risk of rupture for previously ruptured versus 1.3% for unruptured at diagnosis [38]; age at diagnosis (30% increase risk per decade [38], simplified lifetime risk 105 - age in years [49]); drainage pattern (1.6-2.4 fold increase in annual risk for exclusive deep venous drainage [38]); size (controversial, small bAVM presented more often as a rupture in some series [50,51], but not in larger series [8]); location (higher risk with posterior fossa [37]); S-M grade (controversial, some report greater risk with higher grades [35], others show opposite effect [8]); race (non-white having a higher risk [52]); and associated aneurysm (higher risk [44]).

Gross and Du [34] published a meta-analysis of the risk of future hemorrhage and reported an annual risk of hemorrhage for unruptured to be 2.2% (95% CI 1.7-2.7), and for ruptured 4.5% (95% CI 3.7-5.5), in keeping with the spontaneous rupture rate reported by the ARUBA trial [29] 2.2% (95% CI 0.9-4.5). In addition, they confirmed previously known risk factors for rupture, including previous hemorrhage [29,38], exclusive deep venous drainage [38], deep location [37], and associated aneurysm [44]. However, their results have been criticized due to inconsistent characteristics between studies, a low event rate of the subgroup analysis, and a small number at risk for each subgroup after a short period. In addition, the reported 2.2% annual rate of rupture for previously unruptured is slightly higher than other reports, for example, Kim et al. [38] at 1.3% (95% CI 1.0-1.75). This low rate reported by Kim et al. [38] may be due to the potential bias in treating younger patients, thus excluding elderly patients that may have an inherently higher risk of rupture. In contrast, a lack of effect of age may be argued considering that the risk of hemorrhage from the time of birth until the diagnosis is similar to that from the time of diagnosis to hemorrhage and that the most common age at presentation is 25 to 55 years old [45,53]. Thus, consideration of the confidence of age to determine the risk of future hemorrhage is warranted [54]. Also controversial is the presence of an aneurysm. Kim et al. [38] found that beyond the first year, the presence of an aneurysm had a lack of effect on hemorrhage risk, contradicting earlier work [55,56]. Some suggest that earlier work may have been naive to the impact of aneurysms [57]. Beyond five years, some consider the risk of future hemorrhage in previously ruptured equal to unruptured, as the risk of repeat rupture beyond the five years period is negligible [3].

The risk of death from the first hemorrhage ranges from 3% to 58% [50,57-61], with overall morbidity and mortality from hemorrhage reported as a range of 35% to 89% [48,50,55,58,60-62]. This wide range may partially be due to: suboptimal identification of hemorrhage as rupture has been noted at surgery or MRI without any prior clinical event [58]; a very short time interval between first and subsequent hemorrhage may result in subsequent hemorrhage going undetected; patients may die from rupture before arriving at the hospital or confirming diagnosis; any future subarachnoid hemorrhage may be assumed to be from the known bAVM, especially if no other source can be detected by current or available technology; morbidity from bAVM may be indistinguishable from morbidity arising from any early intervention; some data for conservative management hails from a time before current intensive care standards. Thus, the best evidence is from following a case from the time of first detection until the time of first rupture [3]. An adjunct in the assessment of mortality is the indirect method of noting any excess mortality above the norm for that population [62].

Adverse outcomes for surgery are 10% for S-P class A (better than their five- year natural history [31]), 18% for class B (better than their 10-year natural history [31]), and more than 30% for class C [17]. However, the ability to generalize reported surgical outcomes were reviewed and found to be acceptable for S-P class A and B but not acceptable for class C due to varied adverse outcome rate [63]. Factors contributing to surgical risk include location [32], deep venous drainage [32], and diffuse nidus [64,65]. Controversial factors affecting surgical risk include lenticulostriate supply (some report increased surgical risk [66], others [65] report no increased risk possibly due to inadvertent exclusion of lenticulostriate supply from surgery); larger size (reports include both increased risk [32] and no increased risk [64]). Considering these risks, some concerns remain in using only the S-P class to guide management. For example, surgery is recommended for ruptured S-M grade 4 (therefore S-P class C) in patients younger than 20 years with a compact nidus (therefore L-Y grade 4) as the adverse outcome was found to be 10% [39]. In contrast, it is not recommended for a lower S-M grade 2 (S-P class A) in patients older than 40 years with a diffuse nidus (L-Y grade 7) as the adverse outcome was found to be 40% [39]. Hence, the L-Y scale, in addition, to the S-M grade, should be used to assess the risk of surgical intervention.

Drawbacks of radiation include the delay from radiosurgery to obliteration, with reports of increased risk of hemorrhage during this latent period [57]. Prediction of obliteration without neurological deficit can be estimated using the Pollock-Flickinger formula [43]: P-F score = 0.1 x volume (ml) + 0.02 x age (years) + 0.5 (if bAVM located in basal ganglia, thalamus, brainstem). A score ≤1 is approximated to a 90% obliteration rate by 70 months, 70% for scores 1-1.5, 60% for scores 1.5-2, and <50% for scores >2 [43]. Despite repeated radiosurgery and higher radiation doses for larger bAVM, they have reduced obliteration rates with a greater propensity for damage to normal surrounding structures. The best obliteration rates from radiosurgery are for bAVM <3cm with no prior embolization, with at least 60% obliterated within four years [3]. A comparison of radiosurgery and surgery reported that surgery had fewer adverse outcomes than radiosurgery [3], but radiosurgery had fewer operator-dependent outcomes than surgery.

Embolization has been utilized as the sole method of treatment in selected cases and has been reported as most successful for treating S-M grades 1 and 2, with obliteration rates of over 90% for cases that have completed treatment [67,68]. Thus, it may be reasonable to recommend embolization as a first-line treatment for S-P class A, especially when associated with a ruptured aneurysm. However, the ARUBA trial [29] has cautioned the use of solo embolization or embolization combined with radiosurgery, as this was likely the major cause of their adverse outcomes, especially if alternate management options are available and applicable.

Multi-modality strategies have varied combinations and implementations. Preoperative embolization has been shown to vary between surgeons in terms of its usage, vessels targeted, and extent of embolization, and has been reported to decline as surgeons become more confident [67]. Generally, two outcomes are sought. A reduction of volume can be achieved by targeting the nidus. A reduction of intra-operative blood loss may be attempted by targeting feeding vessels. In opposition to preoperative embolization, some describe no increase in adverse outcomes from surgery alone [41,69] and the use of surgical techniques that favor early control of feeding vessels before a deep or circumferential dissection that may help to reduce blood loss [69]. For other combination strategies, evidence is limited. A well-cited source for evidence is the ARUBA trial [29], as discussed. Another study [36] found that planned multi-staged treatment, including focused irradiation had more deaths than cures in their small number of treated patients. Caution is advocated when applying this paper as some patients may have been excluded after the first stage due to various reasons, including complications during the first stage, and these patients would thus have been excluded from progressing to second stage, thereby altering the results of a multi-stage assessment [3]. Cost of treatment has to be considered, with an estimated increase in cost for embolization followed by surgery up to 1.3 to 1.8 times greater than surgery alone [70]. 

The effect of pregnancy on the risk of rupture is controversial; some report increased risk [33,50,71], while others have no increased risk [30,40,72-74]. Thus, firm recommendations cannot be made, and the general trend is to avoid vaginal delivery where possible, delay radiosurgery until after delivery, manage ruptured bAVM as per considerations above while maintaining obstetric principles such as the risk of anti-epileptic medications, positioning to avoid aortocaval compression, and risks of radiation with imaging [3].

**Figure 1:**
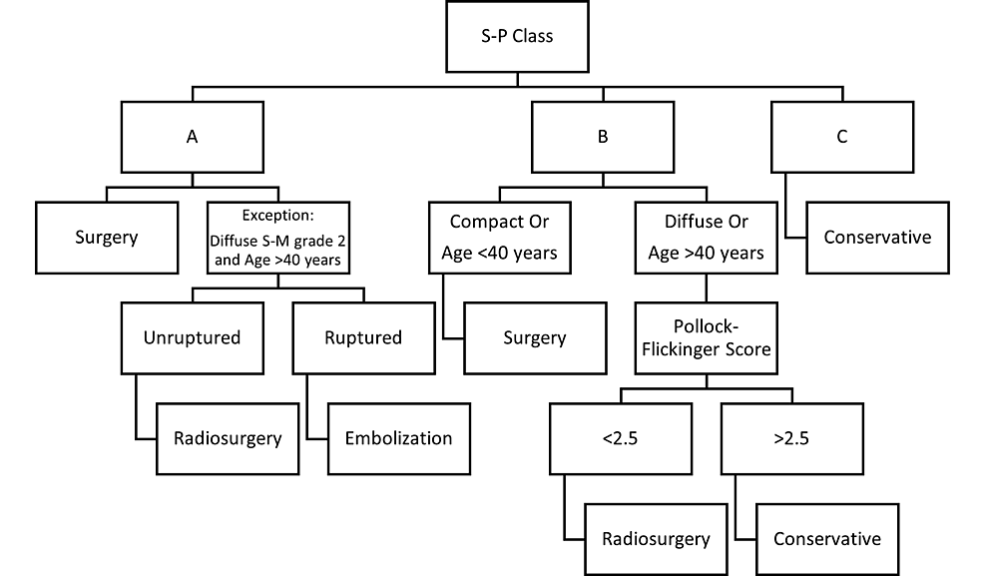
A simplified management algorithm

In Figure 1, the overall management discussed above is synthesized into a simplified flow diagram algorithm.

## Conclusions

Spetzler-Ponce (S-P) class A patients; may be offered surgery if they are good surgical candidates and have a good number of high-quality years of life left. The exception is diffuse Spetzler-Martin (S-M) grade 2 in a patient over 40 years of life: radiosurgery for unruptured and embolization for ruptured. S-P class B may be offered surgery if a compact nidus or if younger than 40 years. If diffuse or age greater than 40, radiosurgery may be offered if the Pollock-Flickinger score is less than 2.5. For the remainder of S-P class B, conservative management may be preferred. S-P class C is generally not treated unless young or those patients with poorly controlled seizures affecting their quality of life are willing to risk permanent neurological deficits. Brain AVM management is a topic that has been studied extensively in the form of low- and middle-class quality evidence. There is a great need for randomized controlled, double-blinded studies from different centers around the world, with meta-analyses of these studies thereafter. Multi-modality treatment in different combinations needs to take a central role in these studies. Further research on the pregnant population needs to occur, with a special strive to attain larger patient numbers. Validation of existing scores in the pediatric population is needed. Careful geographic distribution needs to be considered to include developing nations and studies from non-white majority populations to factor in genetic differences with varying ethnicities. Targeted therapy against genetic mechanisms such as KRAS/BRAF or HOXD3/HOXB3 would have a great clinical impact, especially with some forms of bAVM currently untreatable.
